# Spatiotemporal Patterns of Fish Diversity in the Waters Around the Five West Sea Islands of South Korea: Integrating Bottom Trawl and Environmental DNA (eDNA) Methods

**DOI:** 10.3390/ani15172613

**Published:** 2025-09-05

**Authors:** Young-Ji Yoo, So-Yeon An, Seung-Hwan Lee, Soo-Jeong Lee, Woo-Seok Gwak

**Affiliations:** 1 Department of Marine Biology and Aquaculture, Marine Biology Education and Research Center, Gyeongsang National University, Tongyeong 53064, Republic of Korea; wlduddb01@gnu.ac.kr (Y.-J.Y.); soyeon4525@gnu.ac.kr (S.-Y.A.); 2 Dokdo Fisheries Research Center, East Sea Fisheries Research Institute, National Institute of Fisheries Science, Pohang 37709, Republic of Korea; hwan2915@korea.kr; 3 Climate Environment Resources Division, West Sea Fisheries Research Institute, National Institute of Fisheries Science, Incheon 22383, Republic of Korea; mercione@korea.kr

**Keywords:** demersal fish, fauna, fish behavior, fishery resource, high tidal range, NLL (Northern Limit Line), pelagic fish, Yellow Sea

## Abstract

The waters surrounding the Five West Sea Islands of South Korea are ecologically important but challenging to survey due to their location within a strategic military zone, strong tidal currents, and significant tidal variation. To assess the fish community in this region, we conducted Korea’s first parallel investigation combining traditional bottom trawl surveys with environmental DNA (eDNA) metabarcoding. Sampling was performed at 10 stations in March, May, and August 2023, and the relationship between fish species occurrence and environmental variables (water temperature, salinity, and depth) was examined. Dominant trawl-caught species included *Engraulis japonicus*, *Johnius grypotus*, *Coilia nasus*, and *Okamejei kenojei*, each showing seasonal migration and spawning patterns associated with temperature changes. eDNA analysis detected nine additional species absent from trawl catches, such as *Ilisha elongata* and *Thamnaconus modestus*, demonstrating its sensitivity in identifying both migratory and sedentary taxa. Our findings confirm that eDNA surveys can complement traditional sampling, improving biodiversity assessment in regions with limited accessibility and complex oceanographic conditions.

## Simple Summary

This study presents the first parallel survey using traditional bottom trawling and environmental DNA (eDNA) analysis to investigate fish species around the Five West Sea Islands of South Korea. Over three seasonal sampling periods, fish community composition was closely linked to water temperature changes. The eDNA approach detected both sedentary and transient migratory species that were not recorded in the trawl survey. These results highlight the value of eDNA as a complementary tool to conventional methods, particularly in areas with restricted access, strong currents, or complex environmental conditions.

## 1. Introduction

Effective biodiversity conservation and sustainable fisheries management require accurate assessment of marine resources. In many regions, restricted access, environmental constraints, and geopolitical challenges hinder conventional field survey methods such as trawl sampling. While these methods can determine species composition, they cause environmental disturbance, demand high labor and time, and often have limited temporal and spatial coverage [1]. These issues are particularly acute around the Five West Sea Islands, where dense fishing activity, regulated hours, and political restrictions further complicate surveys [2,3].

Environmental DNA (eDNA) analysis, which detects genetic material shed by organisms into their surroundings, offers a sensitive, non-invasive alternative [4,5]. Widely applied in marine biodiversity monitoring [6,7], eDNA can identify species without direct capture and detect taxa overlooked by conventional gears. In South Korea, eDNA-based ichthyofaunal surveys remain rare, with notable examples in southern coastal waters where eDNA complemented underwater visual census methods [8,9], revealing additional species and validating its utility.

The Yellow Sea, a shallow (<200 m), semi-enclosed marginal sea off Korea’s west coast, maintains a cold-water mass despite lacking a persistent cold current. Cold-water species such as herring (*Clupea pallasii*) and cod (*Gadus macrocephalus*) persist here, but rising sea surface temperatures (SST) threaten these populations; the annual mean SST has increased by 1.44 °C over the past 57 years [10], altering spawning, migration, and community composition.

The Five West Sea Islands—Baengnyeongdo, Daecheongdo, Socheongdo, Yeonpyeongdo, and Udo—experience some of the world’s largest tidal ranges, with semidiurnal tides reaching 10 m [11]. These conditions strongly influence currents, sediments, and habitats [12]. The islands are critical spawning, nursery, and feeding grounds for many commercially and ecologically important species. Previous surveys using trawls, fish traps, and gill nets have reported 12–53 species depending on method and location [13,14,15], but these approaches remain limited by gear selectivity, spatial coverage, and access restrictions.

This study integrates bottom trawl sampling with eDNA metabarcoding to assess fish communities in the waters around the Five West Sea Islands. It represents the first application of this combined approach in the region and provides a framework for biodiversity monitoring in politically sensitive and environmentally challenging marine areas.

## 2. Materials and Methods

### 2.1. Station Designation and Environmental Surveys

**Figure 1:**
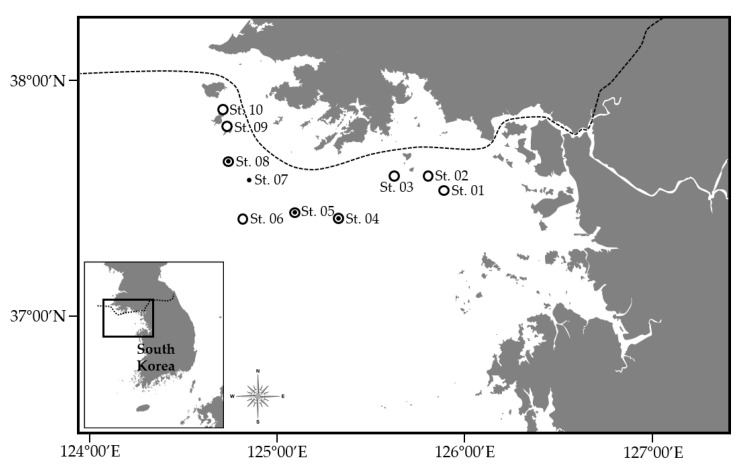
Map showing the study area around the Five West Sea Islands in South Korea. ○ Seawater samples for eDNA analysis. ● Bottom trawl survey.

To investigate the species composition and seasonal variation of fish inhabiting the waters surrounding the Five West Sea Islands, bottom trawl and eDNA surveys were conducted in March, May, and August 2023. A total of 10 sampling stations were established between Yeonpyeongdo and Daecheongdo, taking into account the distance between the islands and the expansion zone of the designated fishing grounds (Figure 1). At each station, the water temperature and salinity were measured using a CTD profiler (Seabird 119).

### 2.2. Bottom Trawl Surveys

Bottom trawl surveys were conducted a total of four times (once each at Stations 4, 5, 7, and 8) in the waters surrounding the Five West Sea Islands using a bottom otter trawl with an end mesh size of 18 mm deployed from the R/V Tamgu 2 (Figure 1).

Resource density (ind./km^2^ or kg/km^2^) was estimated based on catch data and the swept area, incorporating an assumed catch efficiency of 0.5. The swept area was determined from towing distance (or the product of vessel speed and towing duration) and a fixed net width of 12 m, following established protocols [16] (Table 1).

The collected fish species were taxonomically identified with reference to an illustrated book of Korean fishes [17].

### 2.3. Seawater Sampling

For eDNA metabarcoding analysis, 5 L of seawater was collected from a depth of 2–5 m above the seabed at Stations 1–6 and 8–10 in March, May, and August 2023 (Figure 1). To prevent DNA degradation, benzalkonium chloride (BAC) was immediately added to each sample to a final concentration of 0.01%, after which they were stored at −20 °C. The samples were then transported to the Marine Organism Education and Research Center at Gyeongsang National University, where they were filtered using Sterivex filter units (Millipore, Darmstadt, Germany) and stored at −20 °C until further analysis. To prevent cross-contamination between samples during the collection and transportation of the samples, all equipment and work surfaces were disinfected using bleach (active ingredient: sodium hypochlorite). In addition, sterilized water sampling bottles were used for sample collection.

### 2.4. eDNA Extraction

eDNA was extracted from the Sterivex filter units using a DNeasy Blood & Tissue Kit (Qiagen, Hilden, Germany) following a previously described protocol [18] and stored at −20 °C until further analysis. All procedures were carried out in a clean laboratory environment using sterile, disposable equipment. Laboratory benches and instruments were regularly cleaned with 70% ethanol and sterilized using a 10% diluted commercial bleach solution. To assess the potential for cross-contamination between samples during the indoor laboratory procedures, including DNA extraction, a negative control was established by filtering 1 L of deionized–distilled water (DDW) through a Sterivex-HV filter and processing it under identical conditions. No DNA amplification was observed in any of the negative controls, confirming that contamination did not occur. Negative controls were included at every stage of the sampling and laboratory process to continuously monitor for potential cross-contamination. DNA extraction and PCR analysis were also conducted in physically separated laboratory spaces to prevent cross-contamination. DNA purification was conducted using an AMPure XP purification kit (Beckman Coulter, Brea, CA, USA).

### 2.5. Amplicon Library Preparation and MiSeq Sequencing

Amplicon libraries targeting the 12S rRNA gene were constructed using a two-step tailed PCR method with universal fish primers (MiFish U and MiFish E) following a previously reported protocol [19]. The initial PCR mixture (total volume of 10 μL) contained 1.0 μL of 10× Ex Buffer, 0.8 μL of dNTPs (2.5 mM each), 0.5 μL each of 10 μM forward and reverse primers, 2.0 μL of template DNA (max 2 ng/μL), 0.1 μL of ExTaq HS (5 U/μL; TaKaRa, San Jose, CA, USA), and 5.1 μL of DDW. The thermal cycling profile was as follows: 95 °C for 3 min and 35 cycles of 98 °C for 20 s, 65 °C for 15 s, and 72 °C for 20 s, followed by a final extension at 72 °C for 5 min.

The second PCR mixture (total volume of 10 μL) had the same reagent composition, with 2.0 μL of the first PCR product as a template. The thermal cycling profile was as follows: 94 °C for 2 min and 12 cycles of 94 °C for 30 s, 60 °C for 30 s, and 72 °C for 30 s, with a final extension at 72 °C for 5 min.

Amplicon concentrations were quantified using a Synergy H1 reader and the QuantiFluor dsDNA System. Library quality was verified using a Fragment Analyzer with a dsDNA 915 Reagent Kit (Advanced Analytical Technologies, Ankeny, IA, USA). Sequencing was performed on an Illumina MiSeq platform using 2 × 300 bp paired-end reads.

### 2.6. Data Analysis

Read quality filtering was performed using the FASTQ barcode splitter in the FASTX-Toolkit, extracting only sequences that matched the primer sequences exactly [20]. The primer regions and the final 70 bases of each read were trimmed, and reads with quality scores below 20 or lengths under 40 bp were removed using Sickle [21]. Paired-end reads passing quality filters were merged using the Flash tool (v1.2.11) with the following parameters: merged fragment length = 180 bp, read length = 170 bp, and minimum overlap = 10 bp [22].

Operational taxonomic units (OTUs) were clustered at a 97% sequence similarity using USEARCH (v7.0.1090) [23]. Taxonomic assignment of OTUs was conducted using BLAST+ (v2.17.0) searches against the Mitochondrial Genome Database of Fish (MitoFish) with MiFish reference sequences [24].

Non-metric multidimensional scaling (NMDS) was employed using the vegan package (v2.6-10) in R to assess the community similarity among samples. Redundancy analysis (RDA) using Hellinger-transformed species composition data was also conducted to evaluate the relationship between the environmental variables (salinity, depth, and water temperature) and the sampling months (March, May, and August).

## 3. Results

### 3.1. Oceanographic Characteristics

**Figure 2:**
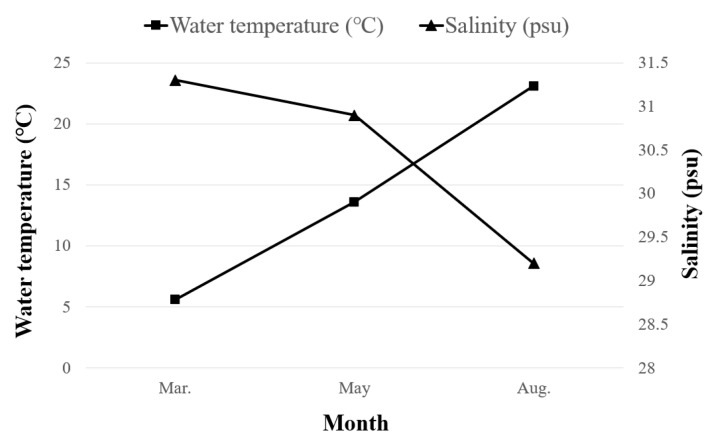
Changes in the average sea temperature (■) and salinity (▲) during the survey period.

During the survey period, the average sea temperature surrounding the Five West Sea Islands ranged from 5.6 °C in March to 23.3 °C in August (Figure 2). The ranges of the SST and sea bottom temperature (SBT) were as follows: 5.4–6.3 °C and 5.1–6.2 °C in March, 11.1–16.4 °C and 10.4–16.4 °C in May, and 19.4–28.0 °C and 13.4–27.0 °C in August (Table 2). These variations are typical of the seasonal changes in temperate marine environments. Salinity was the highest in March and lowest in August, ranging from 27.0 to 31.7 psu (mean 30.5 ± 1.3). In March, May, and August, the measured salinity levels were 30.9–31.7 psu (mean 31.3 ± 0.2), 30.2–31.5 psu (mean 30.9 ± 0.4), and 27.0–31.6 psu (mean 29.1 ± 1.7), respectively. The broadest variation was observed in August.

### 3.2. Fish Species Composition

#### 3.2.1. Bottom Trawl Surveys

**Figure 3:**
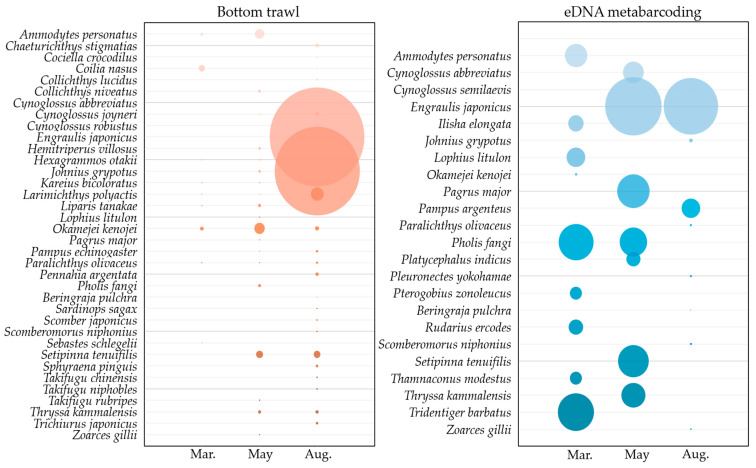
Bubble plot comparing the number of individuals for each fish species identified using bottom trawl surveys (**left**) and the number of reads obtained through eDNA metabarcoding (**right**).

**Figure 4:**
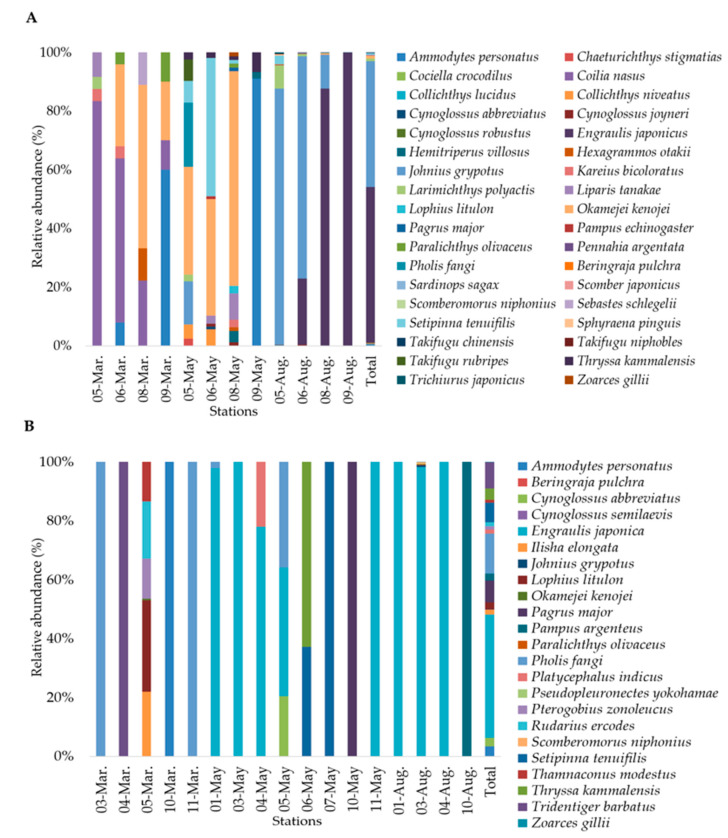
Relative abundance (%) of fish species in March, May, and August at individual sampling stations observed using (**A**) bottom trawl surveys and (**B**) eDNA metabarcoding around the Five West Sea Islands. The number before the month denotes the sampling station.

A total of 857,431 individuals belonging to 36 species, 23 families, and 7 orders were collected using bottom trawling (Figure 3 and Table 3). Perciformes was the most dominant order, accounting for 15 species, followed by Pleuronectiformes, Scorpaeniformes, and Clupeiformes, with 5 species each. The most dominant species over the entire survey period was *Engraulis japonicus* with 455,221 individuals (53.1%), followed by *Johnius grypotus* with 366,758 individuals (42.8%). Other notable species included *Larimichthys polyactis*, *Okamejei kenojei*, and *Setipinna tenuifilis*, with 8361 (1.0%), 7302 (0.9%), and 5072 (0.6%) individuals, respectively (Figure 4A). Of the 36 species identified, *Paralichthys olivaceus*, *L. polyactis*, and *O. kenojei* were observed in all three months, while *Ammodytes personatus*, *Liparis tanakae*, *Pampus echinogaster*, *Kareius bicoloratus*, *J. grypotus*, *S. tenuifilis*, *Chaeturichthys stigmatias*, *Coilia nasus*, *Hexagrammos otakii*, *Cynoglossus joyneri*, and *Thryssa kammalensis* were observed in two of the sampling months.

In March, May, and August, 9 species with 3289 individuals, 21 species with 15,946 individuals, and 23 species with 838,097 individuals were recorded (Table 4). In March, *C. nasus* was the dominant species (54.9%), with *O. kenojei* as the subdominant species (20.2%). In May, *O. kenojei* was dominant (36.2%), followed by *A. personatus* (27.4%). In August, *E. japonicus* was dominant (54.3%), with *J. grypotus* the next most common species (43.7%).

#### 3.2.2. eDNA Surveys

From 135 L of seawater, 1874,813 reads were obtained, with 284,258 (15.2%) assigned to 23 species from 17 families and 7 orders (Figure 3, Table 3). Perciformes had the most species (9), followed by Pleuronectiformes and Clupeiformes (5 each). Only *E. japonicus* and *Pholis fangi* were detected in more than 1 month, and no species were present across all months (Figure 4B). Detected species/read counts by month were March—9 species/80,426 reads; May—7 species/138,732 reads; and August—9 species/65,100 reads (Table 5).

#### 3.2.3. Comparison of the Bottom Trawl and eDNA Surveys

**Figure 5:**
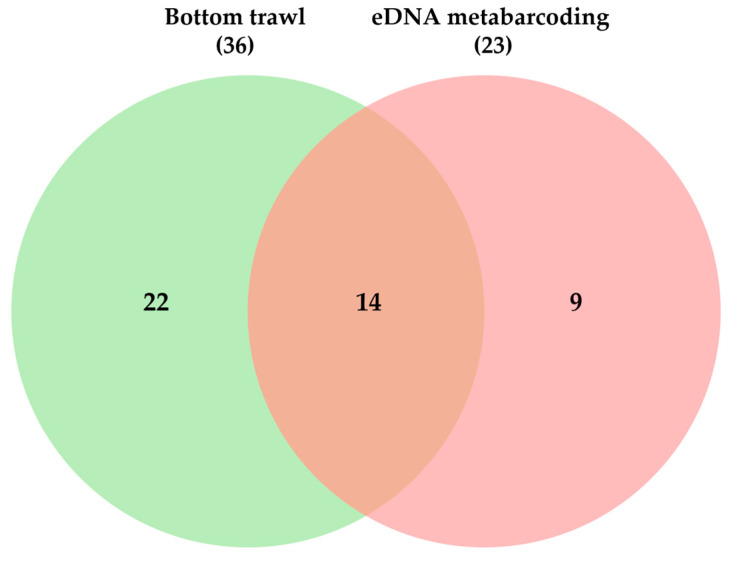
Number of species collected from bottom trawl surveys and eDNA metabarcoding.

Combined, both methods detected 45 species from 25 families and 10 orders (Table 3). Fourteen species were found by both methods, while 22 and 9 were unique to trawling or eDNA, respectively (Figure 5).

### 3.3. Relationship Between Fish Community Similarity and Environmental Factors

**Figure 6:**
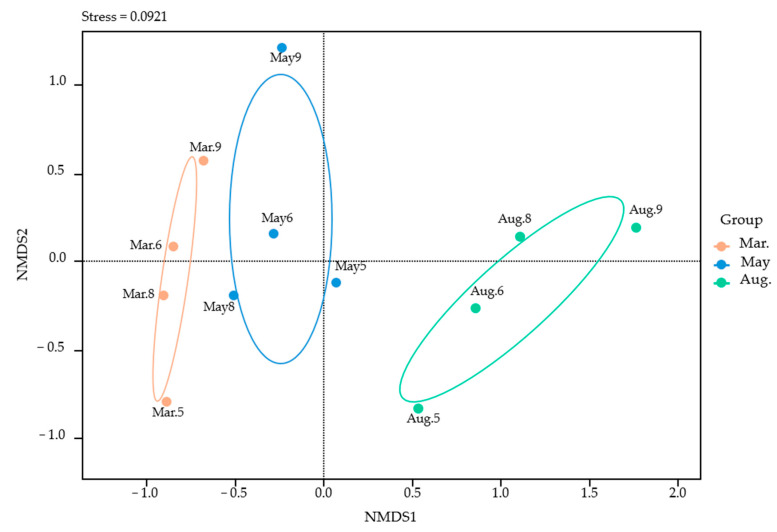
Non-metric multidimensional scaling (NMDS) based on Bray–Curtis similarity (stress = 0.0921). Each point represents an individual sample from March, May, or August. The ellipses denote the 95% confidence interval.

NMDS analysis revealed distinct differences between the March, May, and August groups, indicating temporal shifts in the fish community composition (Figure 6). The March composition was distributed narrowly along the *x*-axis and widely along the *y*-axis, indicating high diversity within the community. The May composition had a relatively wide distribution on both axes, suggesting high overall diversity. The August assemblage was narrowly clustered along the *y*-axis, suggesting possible adaptation to specific environmental conditions.

**Figure 7:**
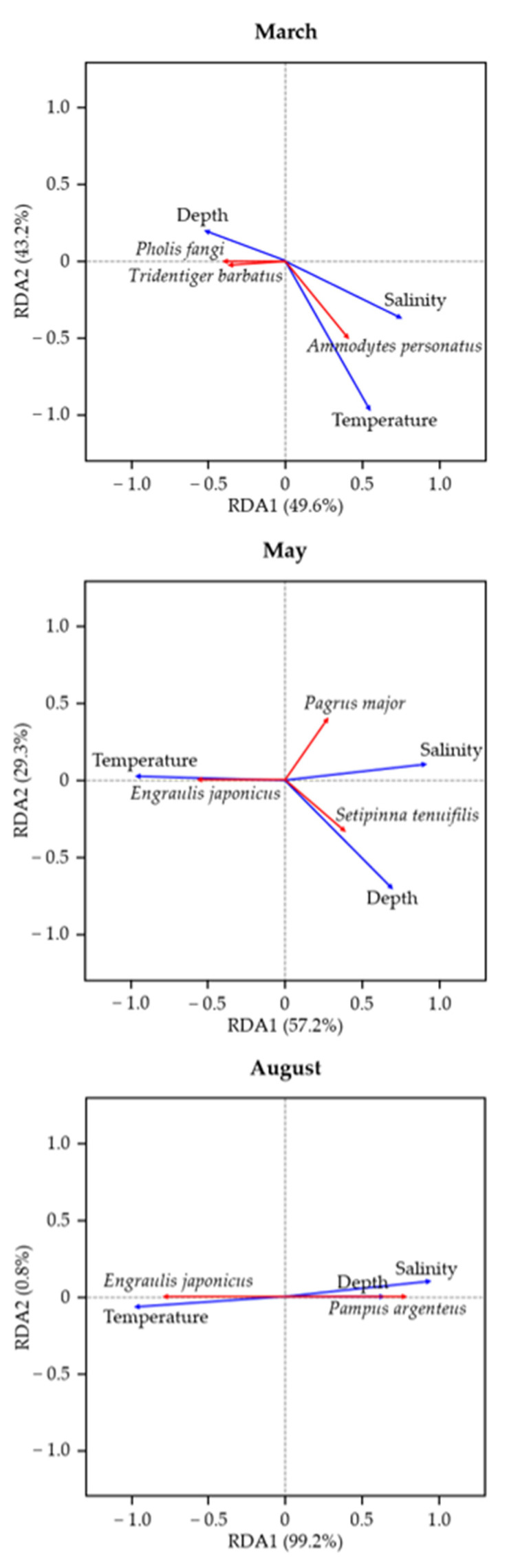
Redundancy analysis (RDA) illustrating the relationships between the dominant species detected using eDNA in March, May, and August and three environmental variables.

The relationship between the dominant species detected in each month and major environmental factors was analyzed using RDA. In March, the RDA1 and RDA2 axes explained 49.6% and 43.2% of the variation, respectively, with salinity and water temperature identified as the most salient environmental factors in the RDA plot (Figure 7). Of the species influenced by the environmental variables, *P. fangi* and *Tridentiger barbatus* were most closely associated with deep and low-water temperature conditions, whereas *Ammodytes personatus* had a strong association with high-water temperature and high-salinity environments. In May, RDA1 and RDA2 explained 57.2% and 29.3% of the variation, respectively, with salinity and depth the most influential environmental factors in the RDA plot (Figure 7). In particular, *E. japonicus* was closely associated with high-water temperature conditions, *Setipinna tenuifilis* had a strong relationship with deeper water, and *Pagrus major* exhibited a distinct correlation with environments characterized by high salinity and deep water. In contrast, water temperature was the most influential environmental factor in August, with RDA1 and RDA2 explaining 99.2% and 0.8% of the variation, respectively (Figure 7). *E. japonicus* exhibited a particularly close association with high-water temperature conditions, whereas *Pampus argenteus* had a strong correlation with high-salinity and deep-water environments.

## 4. Discussion

### 4.1. Fish Diversity and Occurrence Patterns

The high abundance of *Engraulis japonicus* in the trawl catch may reflect migration to spawning grounds, as its spawning season extends from May to September, peaking in June–July [25]. The stations where *E. japonicus* occurred had temperatures (SBT: 15.2 °C; SST: 27.6 °C) within its optimal spawning range (17.0–27.0 °C) [25]. The increased abundance of *Johnius grypotus* in August was likely due to juvenile recruitment after spawning [26], with water temperatures (SBT: 13.4 °C; SST: 28.0 °C) matching its habitat range (12.0–25.4 °C) [27].

In the trawl surveys, *Coilia nasus*, *Okamejei kenojei*, and *E. japonicus* dominated in March, May, and August, respectively. *C. nasus* appeared in large numbers in March but not in May; as a coastal migratory fish inhabiting brackish waters and inner bays, it migrates downstream for spawning from June to July at 15.0–30.0 °C [28,29]. Its absence in May may reflect movement to estuaries once spawning temperatures were reached. For *O. kenojei*, the primary spawning season in the West Sea is thought to occur from May to August [30], possibly explaining the rise in May catches. Its decline by August may be due to migration into deeper, cooler waters during summer high temperatures, reducing activity and catchability [30].

These results indicate that seasonal occurrence patterns of dominant species align with known life histories and temperature preferences, highlighting temperature as a primary factor shaping distribution. Multi-season surveys are therefore essential to capture full community diversity, as single-season sampling risks underrepresenting certain life stages or migratory groups.

### 4.2. Comparison of Bottom Trawling and eDNA Survey Results

Integrating eDNA metabarcoding with trawling provided a broader inventory of the fish community. eDNA detected species not captured in nets, including sedentary taxa (*Pholis fangi*) [25], transient migrants (*Ilisha elongata*), and early life stages of *E. japonicus* [26]. Such detections align with the high sensitivity of eDNA to biological material shed into the environment [4,5].

Although *E. japonicus* was absent in the May trawl survey, its detection via eDNA in both May and August suggests early life stages not captured by nets. In trawls, *E. japonicus* was abundant in August, caught offshore at Stations 5, 6, and 8, whereas eDNA was detected at coastal stations (Figure 1 and Figure 4). This species spawns while migrating north along Korea’s west coast, with eggs concentrated in coastal waters early in the season [25]. As eDNA can detect eggs, larvae, and other post-spawning material [31], detections here likely originated from eggs or larvae. Juveniles migrate offshore after spawning [25], explaining their high trawl occurrence in August.

*J. grypotus* was also dominant in trawls but detected by eDNA only in August. In May, trawls recorded 240 individuals (0.02%) versus 366,518 (42.74%) in August. Low population size can reduce seawater DNA concentration, lowering detection probability [9], likely explaining the May discrepancy. Similarly, *C. nasus* was dominant in March trawls but absent from eDNA, possibly due to its fast-moving migratory behavior [32] and the timing of water collection [33].

*O. kenojei* was abundant in May trawls but detected by eDNA only in March. This may reflect method differences: trawls capture benthic species effectively, whereas eDNA samples were taken 2–5 m above the seabed, reducing detection. In the Yellow Sea, wide tidal ranges and strong currents hinder precise-depth sampling, potentially affecting results. As a cold-water species, *O. kenojei* may also release less DNA due to low activity. For more accurate detection, quantitative PCR with species-specific primers is recommended [34].

Detection of *P. fangi* eDNA in March and May, despite trawl capture only in May, highlights eDNA’s ability to identify species in less accessible habitats. May temperatures (SBT: 10.4–16.4 °C; SST: 16.4 °C) matched reported springtime conditions (16.8 °C) [35]. Given its November–January spawning season [36], individuals likely remained in deeper waters in March, avoiding trawl capture. May occurrence may reflect post-spawning juvenile movement into coastal feeding areas [37]. The March detection despite trawl absence illustrates eDNA’s utility for detecting species in microhabitats beyond the reach of conventional surveys [38].

eDNA-based detection of *I. elongata* exclusively in March aligns with its northward spring migration after overwintering near Jeju Island [39]. Given its spawning period (April–July) [40] and optimal temperature range (20.8–25.5 °C) [41], the cool March conditions (SBT: 5.1 °C; SST: 6.3 °C) suggest detection of transient migrants rather than spawning adults. Considering eDNA’s estimated half-life in temperate marine waters (26 h) [42], the March signal likely reflected recent passage and may serve as a molecular indicator of migratory timing along the west coast.

Detection of *Thamnaconus modestus* eDNA at Station 4 in March under low temperatures (5.1–6.3 °C) contrasts with its preferred range (10.0–28.0 °C) [43]. This may indicate local cold tolerance or eDNA’s sensitivity to low-density or transient individuals. No trawl captures occurred, likely due to habitat mismatch, as survey depths (16 m) were much shallower than its typical range (50–110 m) [43]. Exclusive eDNA detection may reflect vertical segregation or DNA transport from adjacent habitats. Confirmation of its presence will require targeted specimen collection.

Differences between the two methods reflect inherent detection biases. Benthic species like *O. kenojei* may be underrepresented in eDNA, and fast-moving species such as *C. nasus* may be absent from samples taken outside their immediate presence. These results support viewing eDNA and trawling as complementary rather than interchangeable tools.

### 4.3. Changes in the Fish Community Composition Due to Temperature Variation

Temperature was the dominant driver of monthly variation in fish community composition, as shown by NMDS and RDA analyses. Seasonal warming from March to August caused warm-water species to replace cold- or cool-water taxa [10,28], with salinity and depth influencing distribution in certain months [29]. RDA identified *E. japonicus* as the species most sensitive to temperature changes. NMDS1 axis scores arranged chronologically from March to August indicated a gradual seasonal transition in community structure.

The west coast of South Korea experiences strong tides from combined astronomical and meteorological forces [44], complicating station selection. Fixed-point sampling may not fully capture local biodiversity. Future studies should expand station coverage and conduct finer-scale spatial surveys that account for water mass movement by tidal currents.

The region’s strong tidal currents and dynamic water masses likely contribute to fine-scale spatial heterogeneity that fixed-station sampling may not capture [11]. Expanding spatial coverage, integrating hydrodynamic models, and pairing physical with biological data could improve understanding of how oceanographic processes shape community structure.

### 4.4. Advantages and Limitations of eDNA Surveys

This study confirms that eDNA surveys can detect species overlooked by conventional gears, including those in inaccessible habitats, at low densities, or as early life stages [4,5,6,8,9]. eDNA is particularly valuable where fishing operations are physically or politically restricted, as in the Five West Sea Islands. It can also reveal transient migrants and seasonal shifts that gear-limited surveys may miss.

However, eDNA has limitations. False positives may arise from DNA transport by currents [32], and detection can be reduced for species that release little DNA or inhabit unsampled zones such as the seabed [33]. Sampling depth constraints, as in this study, can bias results against benthic taxa. Additionally, eDNA provides presence data but cannot directly estimate abundance without calibration [34].

Combining eDNA with traditional surveys mitigates these issues, pairing eDNA’s broad detection capability with quantitative and size-structured information from captures. This integrated approach enhances biodiversity assessment in sensitive or restricted-access areas, and in the context of climate change, multi-method monitoring will be essential for sustaining biodiversity and fisheries management [10,35].

## 5. Conclusions

Trawling and eDNA surveys around the Five West Sea Islands produced differing species lists, largely due to spawning and migration patterns. These results show that eDNA can reveal diversity obscured by ecological factors, detecting species or life stages missed by nets. However, eDNA may yield false positives from DNA transported by currents, and metabarcoding alone may not fully reflect actual community structure. Increasing survey frequency or spatial coverage can reduce these limitations, making eDNA an effective complement to conventional capture methods. Together, these approaches enable more accurate and rapid assessments in areas with restricted access, support long-term biodiversity monitoring, and allow early detection of invasive species—providing essential information for marine conservation and sustainable fisheries management.

## Figures and Tables

**Table 1:** Parameters used for the calculation of the swept area, including the month, station, average vessel speed (*V*), and towing duration (*T*).

Month	Station	Average Vessel Speed (knot)	Towing Duration (m)
Mar.	05	3.5	30
	06	3.6	30
	08	4.2	30
	09	3.4	30
May	05	4.5	30
	06	3.6	30
	08	4.0	25
	09	3.9	13
Aug.	01	4.0	10
	05	4.6	30
	06	4.2	15
	08	3.7	30
	09	4.1	20

**Table 2:** Environmental conditions in the waters near the Five West Sea Islands measured at individual stations in March, May, and August.

Month	Sampling Station	Water Depth (m)	Surface		Bottom	
			Water Temperature (°C)	Salinity (psu)	Water Temperature (°C)	Salinity (psu)
Mar.	01	7	5.4	31.1	5.3	31.1
	02	10	5.4	30.9	5.3	30.9
	03	17	5.4	30.9	5.3	30.9
	04	16	5.4	31.3	5.3	31.3
	05	48	5.5	31.3	5.1	31.4
	06	52	5.6	31.5	5.3	31.5
	07	42	6.3	31.7	6.2	31.7
	08	21	6.1	31.6	6.1	31.6
	09	11	6.1	31.6	6.1	31.6
	10	37	5.6	31.4	5.7	31.5
May	01	4	14.6	30.8	14.5	30.8
	02	9	16.4	30.3	16.4	30.2
	03	19	16.4	30.3	16.3	30.2
	04	15	15.3	30.7	14.7	30.7
	05	24	14.6	30.9	12.9	31.2
	06	45	15.5	31.1	10.4	31.3
	07	46	15.1	31.1	10.4	31.3
	08	14	13.8	31.3	11.1	31.5
	09	16	11.1	31.5	11.1	31.5
	10	34	11.5	31.4	11.4	31.4
Aug.	01	6	26.1	28.1	26.1	27.9
	02	13	27.2	27.3	27.0	27.1
	03	20	27.0	27.1	27.0	27.0
	04	16	26.3	27.5	25.8	28.1
	05	18	26.2	28.8	25.1	27.1
	06	54	28.0	30.3	13.4	31.6
	07	43	27.6	30.2	15.2	31.4
	08	26	21.1	30.4	15.6	31.3
	09	23	19.4	30.8	19.4	30.8
	10	36	20.7	30.5	18.7	30.9

**Table 3:** Classification of fish species detected using bottom trawl surveys and eDNA metabarcoding in the present study.

Class	Order	Family	Species	eDNA Metabarcoding	Bottom Trawl Surveys
Elasmobranchii	Rajiformes	Rajidae	*Beringraja pulchra*	O	O
	Rajiformes	Rajidae	*Okamejei kenojei*	O	O
Teleostei	Clupeiformes	Alosidae	*Sardinops sagax*		O
	Clupeiformes	Engraulidae	*Coilia nasus*		O
	Clupeiformes	Engraulidae	*Engraulis japonicus*	O	O
	Clupeiformes	Engraulidae	*Setipinna tenuifilis*	O	O
	Clupeiformes	Engraulidae	*Thryssa kammalensis*	O	O
	Clupeiformes	Pristigasteridae	*Ilisha elongata*	O	
	Carangaria incertae sedis	Sphyraenidae	*Sphyraena pinguis*		O
	Eupercaria incertae sedis	Sciaenidae	*Collichthys lucidus*		O
	Eupercaria incertae sedis	Sciaenidae	*Collichthys niveatus*		O
	Eupercaria incertae sedis	Sciaenidae	*Johnius grypotus*	O	O
	Eupercaria incertae sedis	Sciaenidae	*Larimichthys polyactis*		O
	Eupercaria incertae sedis	Sciaenidae	*Pennahia argentata*		O
	Eupercaria incertae sedis	Sparidae	*Pagrus major*	O	O
	Gobiiformes	Gobiidae	*Chaeturichthys stigmatias*		O
	Gobiiformes	Gobiidae	*Pterogobius zonoleucus*	O	
	Gobiiformes	Gobiidae	*Tridentiger barbatus*	O	
	Lophiiformes	Lophiidae	*Lophius litulon*	O	O
	Perciformes	Ammodytidae	*Ammodytes personatus*	O	O
	Perciformes	Hemitripteridae	*Hemitripterus villosus*		O
	Perciformes	Hexagrammidae	*Hexagrammos otakii*		O
	Perciformes	Liparidae	*Liparis tanakae*		O
	Perciformes	Pholidae	*Pholis fangi*	O	O
	Perciformes	Platycephalidae	*Cociella crocodilus*		O
	Perciformes	Platycephalidae	*Platycephalus indicus*	O	
	Perciformes	Sebastidae	*Sebastes schlegelii*		O
	Perciformes	Zoarcidae	*Zoarces gillii*	O	O
	Pleuronectiformes	Paralichthyidae	*Paralichthys olivaceus*	O	O
	Pleuronectiformes	Cynoglossidae	*Cynoglossus abbreviatus*	O	O
	Pleuronectiformes	Cynoglossidae	*Cynoglossus joyneri*		O
	Pleuronectiformes	Cynoglossidae	*Cynoglossus robustus*		O
	Pleuronectiformes	Cynoglossidae	*Cynoglossus semilaevis*	O	
	Pleuronectiformes	Pleuronectidae	*Kareius bicoloratus*		O
	Pleuronectiformes	Pleuronectidae	*Pseudopleuronectes* *yokohamae*	O	
	Scombriformes	Trichiurudae	*Trichiurus japonicus*		O
	Scombriformes	Scombridae	*Scomber japonicus*		O
	Scombriformes	Scombridae	*Scomberomorus niphonius*	O	O
	Scombriformes	Stromateidae	*Pampus argenteus*	O	
	Scombriformes	Stromateidae	*Pampus echinogaster*		O
	Tetraodontiformes	Monacanthidae	*Rudarius ercodes*	O	
	Tetraodontiformes	Monacanthidae	*Thamnaconus modestus*	O	
	Tetraodontiformes	Tetraodontidae	*Takifugu chinensis*		O
	Tetraodontiformes	Tetraodontidae	*Takifugu niphobles*		O
	Tetraodontiformes	Tetraodontidae	*Takifugu rubripes*		O

**Table 4:** Species, number of individuals (N), and biomass (W) of the fish caught using bottom trawl surveys around the Five West Sea Islands of Korea in 2023.

Species	Mar.		May		Aug.		Total	
	N	W	N	W	N	W	N	W
*Ammodytes personatus*	420	19.5	4367	234			4787	253.5
*Chaeturichthys stigmatias*			40	5	694	8	734	13
*Cociella crocodilus*					87	131	87	131
*Coilia nasus*	1860	613.5			39	76	1899	689.5
*Collichthys lucidus*					157	96	157	96
*Collichthys niveatus*			380	151.5			380	151.5
*Cynoglossus abbreviatus*			50	124.5			50	124.5
*Cynoglossus joyneri*			104	68	772	258	876	326
*Cynoglossus robustus*					145	199	145	199
*Engraulis japonicus*					455,221	81,180	455,221	81,180
*Hemitriperus villosus*			269	1224.5			269	1224.5
*Hexagrammos otakii*	42	1	54	3			96	4
*Johnius grypotus*			240	133.5	366,518	16,024	366,758	16,157.5
*Kareius bicoloratus*	101	103	108	901			209	1004
*Larimichthys polyactis*	51	16.5	40	48.5	8270	2262	8361	2327
*Liparis tanakae*	103	9	528	36			631	45
*Lophius litulon*			108	1206			108	1206
*Okamejei kenojei*	666	3962	5778	42,463.5	858	7193.5	7302	53,619
*Pagrus major*			54	1896			54	1896
*Pampus echinogaster*			50	56.5	330	638	380	694.5
*Paralichthys olivaceus*	103	4277.5	54	1414	241	17,190	398	22,881.5
*Pennahia argentata*					520	366	520	366
*Pholis fangi*			360	25			360	25
*Beringraja pulchra*					48	1487	48	1487
*Sardinops sagax*					113	48	113	48
*Scomber japonicus*					193	96	193	96
*Scomberomorus niphonius*					117	147	117	147
*Sebastes schlegelii*	42	0.5					42	0.5
*Setipinna tenuifilis*			2674	1695.5	2398	1534	5072	3229.5
*Sphyraena pinguis*					313	12	313	12
*Takifugu chinensis*					113	668	113	668
*Takifugu niphobles*					117	80	117	80
*Takifugu rubripes*			120	1548			120	1548
*Thryssa kammalensis*			514	79	520	66	1034	145
*Trichiurus japonicus*					313	192	313	192
*Zoarces gillii*			54	72			54	72
Total	3388	9002.5	15,946	53,385	838,097	129,952	857,431	192,339
No. of species	9		21		23		36	

**Table 5:** Species and number of reads (R) of fish detected using eDNA around the Five West Sea Islands of Korea in 2023.

Species	Mar.	May	Aug.	Total
	Read	Read	Read	Read
*Ammodytes personatus*	9763	0	0	9763
*Cynoglossus abbreviatus*	0	8413	0	8413
*Cynoglossus semilaevis*	0	0	13	13
*Engraulis japonicus*	0	61,335	57,691	119,026
*Ilisha elongata*	4775	0	0	4775
*Johnius grypotus*	0	0	238	238
*Lophius litulon*	6712	0	0	6712
*Okamejei kenojei*	118	0	0	118
*Pagrus major*	0	20,740	0	20,740
*Pampus argenteus*	0	0	6850	6850
*Paralichthys olivaceus*	0	0	82	82
*Pholis fangi*	23,606	14,977	0	38,583
*Platycephalus indicus*	0	3778	0	3778
*Pleuronectes yokohamae*	0	0	65	65
*Pterogobius zonoleucus*	2960	0	0	2960
*Beringraja pulchra*	0	0	13	13
*Rudarius ercodes*	4179	0	0	4179
*Scomberomorus niphonius*	0	0	106	106
*Setipinna tenuifilis*	0	18,520	0	18,520
*Thamnaconus modestus*	2919	0	0	2919
*Thryssa kammalensis*	0	10,969	0	10,969
*Tridentiger barbatus*	25,394	0	0	25,394
*Zoarces gillii*	0	0	42	42
Total	80,426	138,732	65,100	284,258
No. of species	9	7	9	23

## Data Availability

All data generated or analyzed during this study are included in this published article.
